# CD70 identifies alloreactive T cells and represents a potential target for prevention and treatment of acute GVHD

**DOI:** 10.1182/bloodadvances.2024012909

**Published:** 2024-07-23

**Authors:** Kriti Verma, Wayne Croft, Sandra Margielewska-Davies, Hayden Pearce, Christine Stephens, Diana Diaconescu, Sarah Bevington, Charles Craddock, Rasoul Amel-Kashipaz, Jianmin Zuo, Francesca A. M. Kinsella, Paul Moss

**Affiliations:** 1 Institute of Immunology and Immunotherapy, University of Birmingham, Birmingham, United Kingdom; 2 Institute of Cancer and Genomic Sciences, College of Medical and Dental Sciences, University of Birmingham, Birmingham, United Kingdom; 3 Centre for Clinical Haematology, Queen Elizabeth Hospital Birmingham, Birmingham, United Kingdom; 4 Warwick Clinical Trials Unit, Warwick University, Coventry, United Kingdom; 5 Pathology, Queen Elizabeth Hospital Birmingham, Birmingham, United Kingdom

## Abstract

Graft-versus-host disease (GVHD) remains a major challenge after allogeneic hematopoietic stem cell transplantation (allo-HSCT), and further understanding of its immunopathology is crucial for developing new treatments. CD70 interacts with CD27 and is upregulated transiently on T cells after recent T-cell receptor (TCR) engagement. Here, we investigated the functional and clinical significance of CD70 expression on T cells during the early posttransplantation period. CD70 was expressed on a subset of highly activated memory T cells within the first 2 weeks after transplant, which then gradually declined in most patients. CD70^+^ T cells exhibited an open chromatin landscape and a transcriptional profile indicative of intense Myelocytomatosis oncogene (*MYC*)-driven glycolysis and proliferation. CD4^+^ and CD8^+^CD70^+^ T-cell numbers increased by ninefold and fourfold, respectively, during acute GVHD (aGVHD) and displayed an oligoclonal TCR repertoire. These cells expressed CCR4 and CCR6 chemokine receptors and were markedly increased in aGVHD tissue samples. Furthermore, CD70^+^ T cells demonstrated alloreactive specificity in vitro, and proliferative and inflammatory cytokine responses were markedly attenuated by CD70 blockade. These findings identify CD70 as a marker of highly activated alloreactive T cells and reveal the potential therapeutic importance of inhibiting CD27-CD70 costimulation in both the prophylaxis and treatment of aGVHD.

## Key Points

• Proinflammatory CD70^+^ T cells develop after HSCT and are enriched in blood and tissue at the time of aGVHD.
• Blockade of CD70 on T cells reduces alloantigen-stimulated T-cell proliferation and cytokine secretion.

## Visual Abstract

**Figure:**
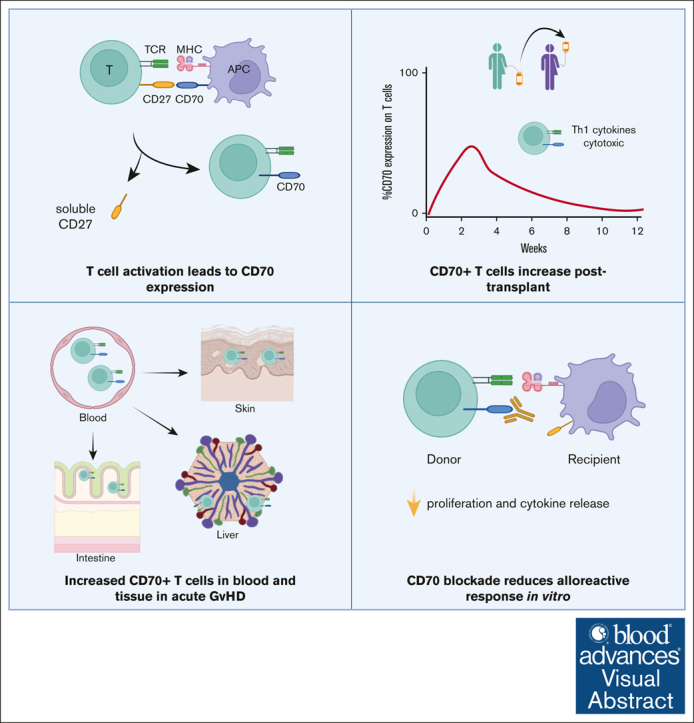

## Introduction

Allogeneic hematopoietic stem cell transplantation (allo-HSCT) is used widely for the treatment of hematological malignancies, bone marrow failure syndromes, and genetic disorders.^1^ Despite its efficacy, the development of acute graft-versus-host disease (aGVHD) remains a significant complication, necessitating immunosuppressive prophylaxis that compromises antitumor and antipathogen immune responses.^2^^,^^3^ Although the prevalence of aGVHD varies according to conditioning regimen and patient demographics, rates of grade 2 to 4 aGVHD approach 50% in most cohorts, and ∼15% of patients experience severe grade 3 to 4 aGVHD despite prophylactic measures.^4^

The pathogenesis of aGVHD is driven primarily by the activation of donor T cells through allogeneic recognition of minor histocompatibility antigens.^5^, ^6^, ^7^ This immune response can mediate tissue damage and dysfunction in a variety of target organs, making GVHD a major contributor to morbidity and mortality in allo-HSCT.^8^^,^^9^ CD70, a member of the tumor necrosis factor family, is a costimulatory molecule that is absent on resting lymphocytes but expressed after activation.^10^, ^11^, ^12^ Engagement of CD70 with its sole receptor CD27 plays a crucial role in T-cell activation^13^, ^14^, ^15^ and has been implicated in autoimmune diseases such as rheumatoid arthritis and systemic lupus erythematosus.^16^

Building upon previous research that identified CD70 messenger RNA upregulation in T cells after allogeneic activation^6^ and considering murine studies suggesting the potential of CD70 as a determinant of GVHD,^17^^,^^18^ we investigated the functional relevance, transcriptional profile, and epigenetic regulation of CD70-expressing T cells in patients undergoing allo-HSCT.

## Materials and methods

### Patient sample collection and preparation

Patients who underwent allo-HSCT or autologous HSCT for hematological malignancies at the Birmingham Centre for Cellular Therapy and Transplantation were enrolled in the study after obtaining full written informed consent (05/Q2707/175). Patient characteristics are shown in Table 1 and supplemental Table 2. All patients received ciclosporin-based GVHD prophylaxis. Antimicrobial and viral monitoring were carried out according to standard and consistent institutional policies. Classification of aGVHD was according to the Mount Sinai Acute GVHD International Consortium (MAGIC) criteria.^19^ Patients who exhibited grade 2 to 4 classical aGVHD were grouped as GVHD, and patients with no or grade 0 to 1 GVHD were grouped as non-GVHD. Ten milliliter to 50-mL peripheral blood was collected on the day of the transplant before infusion of the stem cells and then every 2 weeks until first 3 months, at onset of aGVHD (sampled at diagnosis prior to treatment), and monthly for up to 2 years. After receipt of the stem cell graft by patients, residual graft T cells from patients were retrieved from the stem cell bag (SCB) by flushing with phosphate-buffered saline. Peripheral blood mononuclear cells (PBMCs) were isolated by density gradient centrifugation and frozen in fetal calf serum (FCS) containing 10% dimethyl sulfoxide and stored in liquid nitrogen for later use. Serum collected at each time point was stored at –20°C. GVHD histopathology-confirmed formalin-fixed parafin embedded tissue sections were obtained from Human Biomaterials Resource Centre, Birmingham, and used for hematoxylin and eosin and immunofluorescence staining. Four millimeter skin puncture biopsies were collected in RPMI plus 10% FCS and digested immediately for flow cytometry as described earlier.^20^

**Table 1.** **Patient characteristics**

Characteristic	Feature	Total	ATAC-seq	RNA-seq	TCR-seq
		N = 88	n = 7	n = 4	n = 5
Age	Median (range)	53 (19-72)	42 (19-73)	50 (37-66)	37 (33-66)
Sex	Male (%)	56 (64)	5 (71)	4 (100)	4 (80)
	Female (%)	32 (36)	2 (29)	0	1 (20)
Disease	AML (%)	35 (40)	2 (29)	2 (50)	3 (60)
	MDS (%)	21 (24)	1 (14)	0	0
	CML (%)	3 (3)	0	0	0
	Myelofibrosis (%)	1 (1)	0	1 (25)	0
	ALL (%)	9 (10)	3 (43)	0	2 (40)
	NHL (%)	13 (15)	1 (14)	1 (25)	0
	HL (%)	4 (5)	0	0	0
	CLL (%)	1 (1)	0	0	0
	T-PLL (%)	1 (1)	0	0	0
	D^+^/R^+^ (%)	32 (36)	2 (29)	3 (75)	1 (20)
	D^+^/R^–^ (%)	3 (3)	0	0	0
	D^–^/R^+^ (%)	21 (24)	3 (43)	0	3 (60)
CMV	D^–^/R^–^ (%)	32 (36)	2 (29)	1 (25)	1 (20)
	Reactivation (%)	21 (24)	0	0	1 (20)
EBV	Reactivation (%)	9 (10)	1 (14)	1 (25)	1 (20)
Conditioning	Cy/TBI (%)	10 (11)	3 (43)	0	2 (40)
	Flu/Bu/ATG (%)	18 (21)	1 (14)	3 (75)	2 (40)
	Flu/Mel/Campath (%)	33 (38)	2 (29)	0	0
	Flu/Bu/Camptah (%)	1 (1)	0	0	0
	Bu/Cy (%)	3 (3)	1 (14)	0	1 (20)
	BEAM/Campath (%)	8 (9)	0	1 (25)	0
	Flu/Cy/TBI (%)	5 (6)	0	0	0
	FLAMSA/Bu (%)	10 (11)	0	0	0
GVHD prophylaxis	Ciclosporin only (%)	58 (66)	4 (57)	4 (100)	2 (40)
	Ciclosporin + methotrexate (%)	15 (17)	3 (43)	0	3 (60)
	Ciclosporin + MMF (%)	15 (17)	0	0	0
Source of graft	PBSC (%)	83 (94)	6 (86)	4 (100)	3 (60)
	BM (%)	3 (3)	1 (14)	0	2 (40)
	Cord (%)	2 (2)	0	0	0
Type	MUD (%)	62 (71)	5 (71)	3 (75)	3 (60)
	MRD (%)	23 (26)	2 (29)	1 (25)	2 (40)
	Cord (%)	3 (3)	0	0	0
GVHD	Acute (2-4) (%)	31 (35)	7 (100)	4 (100)	5 (100)
	Chronic (%)	9 (10)	1 (14)	0	0
	Median day of onset	+50	+98	+117	+70
Relapse (%)		13 (15)	0	1 (25)	0

ALL, acute lymphoblastic leukemia; AML, acute myeloid leukemia; ATG, antithymocyte globulin; BEAM, BCNU, etoposide, cytarabine and melphalan; BM, bone marrow; Bu, Busulfan; CLL, chronic lymphocytic leukemia; CML, chronic myeloid leukemia; CMV, cytomegalovirus; Cy, cyclophosphamide; D, donor; EBV, Epstein-Barr virus; FLAMSA, fludarabine, amsacrine, and cytarabine; Flu, fludarabine; HL, Hodgkin lymphoma; MDS, myelodysplastic syndrome; Mel, melphalan; MMF, mycophenolate mofetil; MRD, measurable (or minimal) residual disease; MUD, matched unrelated donor; NHL, non-Hodgkin lymphoma; PBSC, peripheral blood stem cells; R, recepient; TBI, total body irradiation.

The studies involving humans were approved by the National Research Ethics Service Committee West Midlands, South Birmingham (BOOST 15/WM/0194). The studies were conducted in accordance with the local legislation and institutional requirements.

### Phenotypic analysis

Cells were thawed using RPMI supplemented with 10% FCS and resuspended in magnetic-activated cell sorting buffer. Surface staining was performed with pretitrated antibodies (supplemental Table 1) on ice for 30 minutes in dark. Unstained or fluorescence-minus-one cells were used as controls. Propidium iodide or Fixable Viability dye (Invitrogen) was added immediately before acquisition to exclude dead cells. Data were acquired on a Gallios flow cytometer, followed by analysis using Kaluza software (both Beckman Coulter).

### ATAC-seq

CD70^+^ and CD70^–^ T-cell subsets were sorted from cryopreserved PBMCs of 7 patients with aGVHD sampled within 0 to 7 days before aGVHD diagnosis. Assay for transposase-accessible chromatin with sequencing (ATAC-seq) library preparation and analysis were performed as described previously.^21^^,^^22^ The libraries were quantified using Qubit fluorometer (Life Technologies), and the size was determined using TapeStation (Agilent). Sequencing was performed on high-throughput benchtop NextSeq 500/550 Sequencer using NextSeq 500/550 High-Output Kit v2-75 cycles (Illumina). Detailed method on ATAC-seq data analysis is provided in the supplemental Data.

### RNA-seq

Total RNA was isolated using miRNeasy mini prep kit (Qiagen) from sorted CD70^+^ and CD70^–^ T cells from cryopreserved PBMCs of 5 patients with aGVHD sampled within 0 to 7 days before aGVHD diagnosis using FACSMelody (BD) according to the manufacturer’s protocol. QuantSeq 3’ messenger RNA-seq (Lexogen) and Immune Repertoire sequencing (Qiagen) kit was used to perform transcriptional profiling and T-cell receptor (TCR) sequencing, respectively. Libraries were prepared according to the manufacturer’s protocol using 10-ng total input RNA and quantified using Qubit fluorometer. Detailed method on RNA sequencing (RNA-seq) and TCR sequencing data analysis is provided in the supplemental Data.

### Enzyme-linked immunosorbent assay

Human CD27 Duoset enzyme-linked immunosorbent assay **(**DY382-05; R&D systems) was used to quantify the concentration of soluble CD27 in the serum of patients who underwent allo-HSCT using the manufacturer’s protocol. The concentration was measured against a standard curve using 4-parameter logistic curve fit.

### T-cell function assays

Cryopreserved PBMC samples from patients who developed aGVHD after transplantation and their patient pretransplantation PBMC samples were seeded in 24-well cell culture plates and rested overnight at 37°C to allow for cells to recover. PBMCs were harvested the next day, and T cells were depleted from pretransplantation samples using positive magnetic bead selection (Miltenyi Biotec). T cells were sorted from posttransplantation PBMCs to a purity of >98%, and responses to T-cell–depleted pretransplantation patient PBMCs were measured in triplicate and seeded at a concentration of 10^4^ T cells per well at a 1:2 ratio with stimulator PBMCs in a mixed lymphocyte reaction. Purified anti-CD70 (10 μg/mL; clone 113-16, BioLegend) was used for blocking.^23^ For measuring cell proliferation, posttransplant T cells were labeled with 2.5-μM carboxyfluorescein diacetate succinimidyl ester (BioLegend) at 37°C for 20 minutes, stopped by the addition of growth media and 2 minutes on ice, before adding to the mixed lymphocyte reaction. The cells were cultured for 5 days at 37°C. For interferon gamma (IFN-γ) enzyme-linked immunospot (ELISPOT), cells were seeded on a multiscreen 96-well plate (Millipore) coated with IFN-γ capture antibody (Mabtech) at 4°C overnight. For background spot determination, negative controls included wells containing dimethyl sulfoxide and patient pretransplantation PBMCs. ELISPOT plates were incubated for 16 to 18 hours at 37°C and developed as per the manufacturer’s instructions (Mabtech). IFN-γ spots were counted using an Advanced Imaging Devices automated ELISPOT reader.

### Immunofluorescence staining

Immunofluorescence staining was performed using Opal 3-Plex kit (Perkin Elmer). Sections of paraffin-embedded tissue (aGVHD patient skin, n = 6; aGVHD patient gut, n = 4; aGVHD patient liver, n = 2; healthy volunteer normal skin, n = 2; and tonsil, n = 1) were cut to a thickness of 4 μm. The sections were heat treated, then dewaxed and dehydrated, followed by blocking of endogenous peroxidase activity in 0.3% H_2_O_2_. Citrate buffer antigen retrieval method was performed (pH 6), and slides were blocked in horse serum (Vector Laboratories). Primary antibodies were applied (CD70 [Proteintech; clone 1A11A10] and CD3 [Invitrogen; clone OKT3]), and slides were incubated overnight. ImmPRESS horseradish peroxidase reagent Universal Anti-Mouse/Rabbit immunoglobulin G (Vector Laboratories) secondary antibody was added. For visualization, Opal tyramide signal amplification fluorescent dyes were used (Fluorescein, Cyanine3; Perkin Elmer) according to the manufacturer’s protocol. Sections were counterstained with DAPI (4′,6-diamidino-2-phenylindole) and imaged using a Nikon E600 UV microscope.

## Results

### CD70^+^ T cells are recently activated effector cells and increase early after allogeneic stem cell transplantation (SCT)

**Figure 1.**
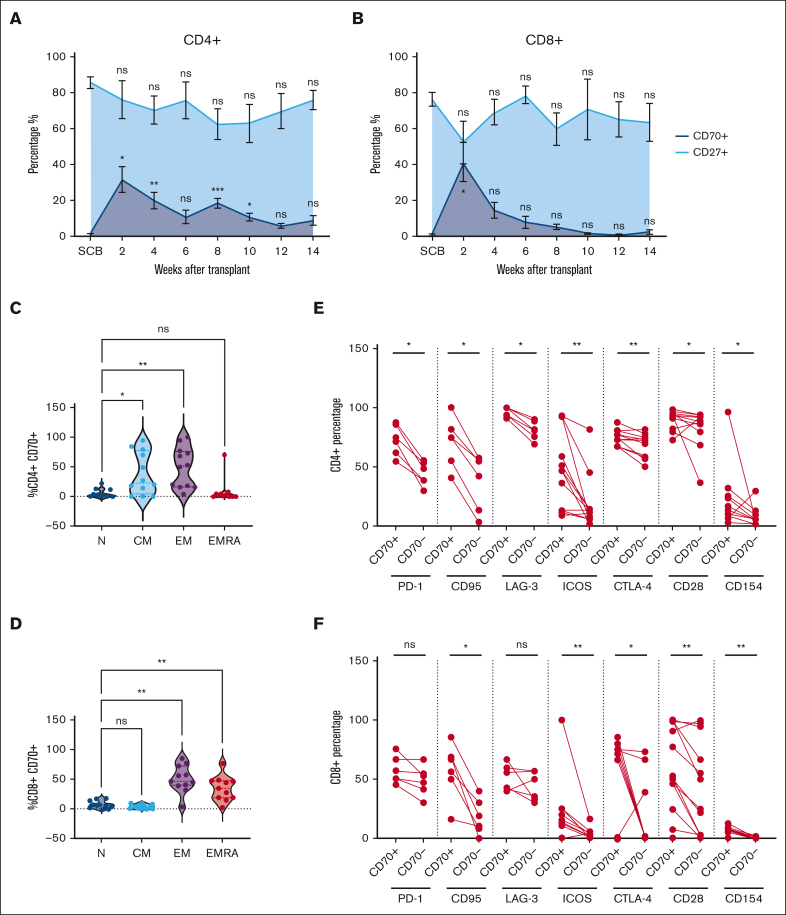
**Frequency and phenotype of CD70^+^ T cells in blood after allo-SCT.** (A-B) Frequency of circulating CD70^+^ (dark blue) and CD27^+^ (light blue) T cells in the blood at 2-week intervals after allo-HSCT during early immune reconstitution until 14 weeks (3 months, n = 14) within the CD4^+^ (A) and CD8^+^ (B) T-cell compartments. Distribution of naïve (N), central memory (CM), effector memory (EM), and EM-like CD45RA^+^ EMRA cells within the CD4^+^CD70^+^ (n = 11) (C) and CD8^+^CD70^+^ (n = 11) (D) T cells, identified using cell surface expression of CD45RA and CCR7. (E-F) Cell surface expression of PD-1 (n = 6), CD95 (n = 6) and LAG-3 (n = 6), ICOS (n = 13), CTLA-4 (n = 13), CD28 (n = 13), and CD154 (n = 13) on CD70^+^ and CD70^–^ population as percentages within the CD4^+^ (E) and CD8^+^ (F) T-cell populations in blood at 2 weeks (day 14) after allo-HSCT. Statistical comparisons were performed using 1-way analysis of variance (ANOVA) with Dunn multiple comparison test (A-D) or Wilcoxon matched-pairs signed-rank test (E-F): ns, not significant; ∗*P* < .05; ∗∗*P* < .01; ∗∗∗*P* < .005. CTLA-4, Cytotoxic T-lymphocyte associated protein 4; EMRA, Terminally differentiated effector memory cells re-expressing CD45RA.

The frequency of CD27^+^ and CD70^+^ T cells after allo-HSCT was assessed by flow cytometry (supplemental Figure 1A). Analysis was undertaken on the donor SCB and patient blood samples taken at 2-week intervals up to 14 weeks after cell infusion (Figure 1A-B; Table 1). CD70^+^CD4^+^ T cells represented a median of 1.1% of T cells in the SCB, but this increased markedly to 33% at day 14 (2 weeks after HSCT; *P* < .05; Figure 1A). Similarly, CD70 expression on CD8^+^ T cells increased from 0.6% in the SCB to 34% at day 14 (*P* < .05; Figure 1B). However, the trajectory of CD70 expression on CD4^+^ and CD8^+^ T cells diverged from this time point. CD70 expression on CD4^+^ T cells remained elevated at 16% and 17% at weeks 4 and 8, respectively, whereas on CD8^+^ T cells, it declined steadily from day 14, reaching 11% and 3% at weeks 4 and 8. The observed fluctuations in CD70^+^ T cells inversely correlated with the percentage of CD27^+^ cells, suggesting a mutually exclusive expression pattern on T cells (Figure 1A-B, respectively). Moreover, the substantial increase in CD70^+^ T cells at day 14 after transplant exceeded the values observed in autograft transplants (supplemental Figure 1B).

CD70^+^ T cells exhibited an antigen-experienced phenotype, and CD4^+^ populations resided predominantly in the effector (51%) and central memory populations (23%), whereas CD8^+^ subsets were divided between effector (48%) and effector-CD45RA (35%) cells (Figure 1C-D). Of note, CD4^+^CD70^+^ T cells consistently lacked FOXP3 expression (supplemental Figure 1D). Extended analysis of CD4^+^CD70^+^ T cells revealed heightened expression of the Fas receptor (CD95), indicative of recent TCR engagement (75% vs 49% on CD4^+^CD70^–^ T cells). Expression of checkpoint proteins Programmed cell death protein 1 (PD-1), Lymphocyte-activation gene 3 (LAG-3), and Cytotoxic T-lymphocyte associated protein 4, (CTLA-4) was also increased (73%, 97%, and 74% vs 44%, 81%, and 71%, respectively; Figure 1E). Activation markers Inducible T-cell costimulator (ICOS) and CD40L were elevated by 3.6-fold (47% vs 13%) and 2.4-fold (13% vs 5.4%), respectively. CD8^+^CD70^+^ T cells also displayed strong upregulation of Fas (62% vs 15% on CD8^+^CD70^–^ T cells), CTLA-4 (71% vs 0.7%; *P* ≤ .01 median), and costimulatory molecules ICOS, CD28, and CD40L (12%, 53%, and 4.4% vs 1.5%, 25%, and 0.35%, respectively; *P* ≤ .01). In contrast, no differences in PD-1 and LAG3 expressions were seen (Figure 1E-F). These findings indicate that CD70^+^ T cells display features of recent TCR engagement, are highly activated, and peak early in the posttransplant period.

### CD70^+^ T cells show increased regions of open chromatin and a transcriptional signature of proliferation at the time of GVHD

**Figure 2.**
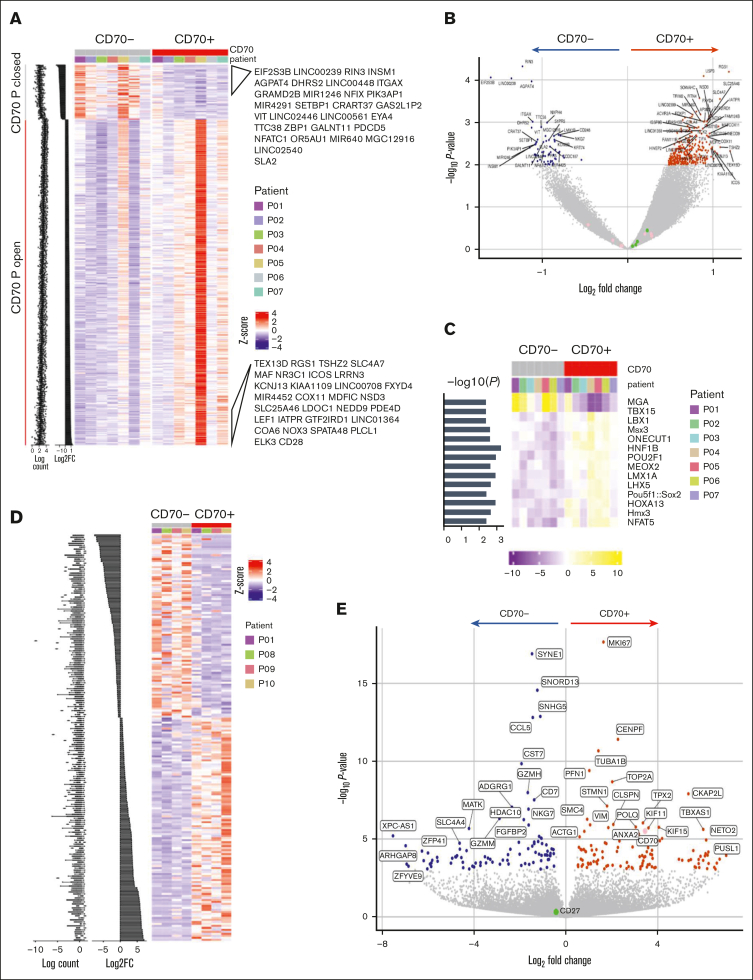
**Chromatin accessibility and transcriptional profile of CD70^+^ T cells in GVHD.** (A) Profile of differentially accessible chromatin (DAC) loci between sorted CD70^+^ and CD70^–^ T cells from the blood of patients with aGVHD (n = 7). DACs were ranked by CD70^+^ vs CD70^–^ fold change, and gene labels represent the loci-associated gene annotations for the top 30 CD70^+^ open and CD70^+^ closed sites. (B) Pairwise comparisons of chromatin accessibility at peak regions. The x-axes indicate Log_2_ fold change, and the y-axes indicate unadjusted –Log_10_*P* value of all peaks. Colored points indicate DAC sites, with inaccessible sites as blue and accessible sites as red. (C) TF motif scores are shown in the heat map plot, based on the motifs of TFs enriched within the differentially accessible peaks in each sample. (D) Profile of differentially expressed genes (DEGs) between CD70^+^ and CD70^–^ T cells from blood of patients with aGVHD (n = 4) ranked by fold change. (E) Pairwise comparison of gene expression in CD70^+^ vs CD70^–^ cells showing –Log_2_*P* value vs Log_2_ fold change of all genes. Colored points indicate DEGs that are downregulated (blue) and upregulated in CD70^+^ (red).

Using ATAC-seq, chromatin accessibility was compared between CD70^+^ and CD70^–^ T cells (Table 1; supplemental Figure 2A). Chromatin regions were relatively more open in CD70^+^ cells than in the CD70^–^ subset, indicating their increased transcriptional potential (Figure 2A). Of note, the CD70 chromatin locus remained accessible within CD70^–^ cells. Genes associated with open peaks included *RGS1*, implicated in autoimmune conditions,^24^ and *USP3*, which suppresses type I IFN signaling^25^ (*P* < .01; Figure 2B). The relative enrichment of transcription factor (TF) binding motifs in ATAC-seq data revealed that TF binding motifs for *MGA* (MYC associated factor X gene–associated protein) and *T-BOX1* were differentially closed in CD70^+^ T cells, whereas the binding motifs for *NFAT5*, *POU2F1*, and *POU5F1* became accessible (Figure 2C). Enrichment analysis identified cytokine production, T-cell differentiation, and lymphocyte activation as biological processes to be enriched with CD70^+^ open differentially accessible chromatin regions compared with CD70^–^ subset (supplemental Figure 2B).

The transcriptional profile of sorted CD70^+^ and CD70^–^ T cells was determined by RNA-seq (Table 1; supplemental Figure 2C), and gene set enrichment analysis confirmed that most highly expressed genes overlapped with the differentially accessible peaks on the chromatin (supplemental Figure 2D). A total of 112 genes were upregulated in CD70^+^ cells, including CD70, whereas 92 genes were downregulated (Figure 2D-E). Notably, several *MYC* target genes associated with cell cycle–related signaling pathways, including G2/M checkpoint, E2F targets, and the mitotic spindle, were considerably enriched in CD70^+^ T cells, indicating high levels of proliferation (supplemental Figure 2E). Glycolysis and inflammatory response pathways were also enriched together with T-cell effector genes such as microtubule nucleation factor *TPX2*.^26^

### CD70^+^ T cells are oligoclonal and not driven by pathogen recognition

**Figure 3.**
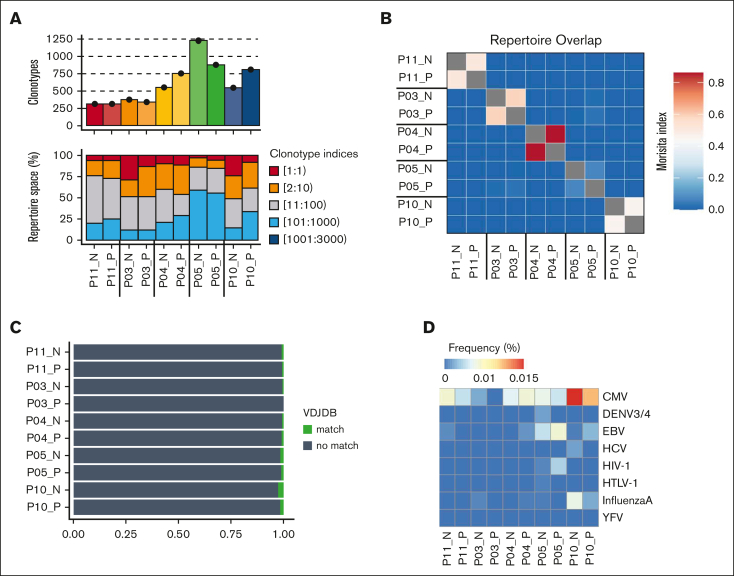
**TCR-α chain immune repertoire sequencing of sorted CD70^+^ and CD70^–^ T cells from 5 patients with aGVHD.** (A) Samplewise number of unique clonotypes identified (top) and percentage of the total repertoire occupied by TCR sequence groupings by index (bottom). TCR sequences were ranked by abundance, with index 1 being the most abundant. (B) Repertoire overlap assessed by the Morisita index. (C) Sample wise tracking of the top 5 clonotypes in each CD70^+^ sample. Shadings indicate shared CDR3 amino acid sequence between samples. (D) Profile of antigen specificity frequencies from all clonotypes that mapped to known public CDR3 amino acid sequences in the variable diversity joining (VDJ) database. VDJDB, VDJ database.

The relative clonality of T-cell populations is valuable in assessing the breadth of antigen engagement and proliferative expansion. As such, we determined the TCR repertoire of CD70^+^ and CD70^–^ T cells within blood collected from 5 patients sampled at 0 to 7 days before aGVHD diagnosis (median aGVHD onset +70 days after transplant; Table 1). CD70^+^ and CD70^–^ T-cell populations demonstrated comparable clonality, each with an average of 300 to 1220 clonotypes (Figure 3A; supplemental Figure 3A). Of note, the top 10 most abundant clones comprised 25% to 50% of the T-cell repertoire in most cases, indicating an oligoclonal TCR repertoire and low repertoire diversity (supplemental Figure 3B). No difference was seen in the number of unique clonotypes or diversity between CD70^+^ and CD70^–^ T cells (supplemental Figure 3C).

We next investigated the sharing of TCR clonotypes between the 2 populations. Interestingly, some overlap of T-cell clones between CD70^+^ and CD70^–^ T cells was seen in each individual patient, but minimal sharing of TCR sequences was observed between different patients (Figure 3B). The top 5 most expanded dominant clonotypes of the CD70^+^ T-cell population for each patient are depicted in supplemental Figure 3D. To explore the potential antigenic specificity of expanded CD70^+^ T-cell clones, we annotated TCR clonotypes with sequences from well-defined antigenic specificity within variable diversity joining databases. Less than 2% of clones exhibited specificity for viral pathogens such as cytomegalovirus or Epstein-Barr virus (Figure 3D). In contrast, the top 10 most abundant clones in each sample did not have a TCR match, suggesting that they likely represent alloreactive T cells with specificity for targets such as minor histocompatibility antigens (supplemental Figure 3D).

### CD70^+^ T cells are increased in blood and tissues during aGVHD

**Figure 4.**
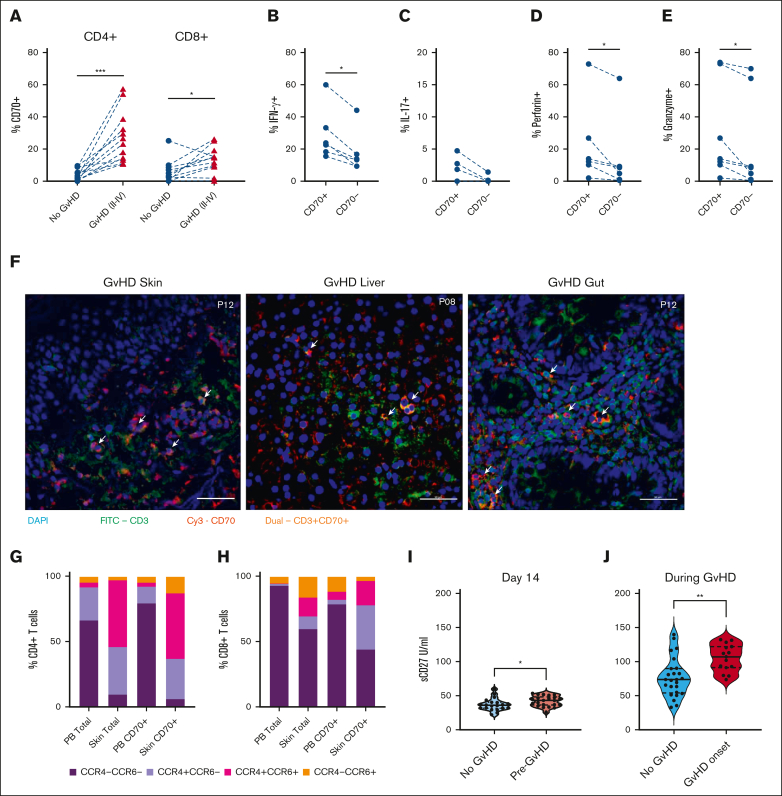
**Effector CD70^+^ T cells are increased in blood and skin of patients with GVHD.** (A) Percentage of CD4^+^CD70^+^ (left) and CD8^+^CD70^+^ (right) cells in patients who underwent allo-HSCT, with ongoing GVHD (n = 12) vs those without aGVHD (no GVHD; n = 12) at matched time points after HSCT. (B) Percentage of IFN-γ^+^ cells within CD4^+^CD70^+^ and CD4^+^CD70^–^ cells in blood of patients with ongoing aGVHD (n = 6). (C) Percentage of interleukin-17–positive (IL-17^+^) cells within CD4^+^CD70^+^ and CD4^+^CD70^–^ cells in blood of patients with ongoing aGVHD (n = 6). (D) Percentage of perforin-positive cells within CD4^+^CD70^+^ and CD4^+^CD70^–^ cells in blood of patients with ongoing aGVHD (n = 6). (E) Percentage of granzyme-B–positive cells within CD4^+^CD70^+^ and CD4^+^CD70^–^ cells in blood of patients with ongoing aGVHD (n = 6). (F) Immunofluorescence staining on skin, gut, and liver FFPE sections from 4 patients with severe aGVHD after allo-HSCT. Staining was performed with nuclei stain DAPI (blue), CD70-Cy3 (red), and CD3-FITC (green). Images taken at 40× magnification. Arrows indicate presence of double-positive (CD3^+^CD70^+^) T cells. (G-H) Cell surface expression of chemokine receptors CCR4 and CCR6 on total CD4^+^ and CD4^+^CD70^+^ (G), and total CD8^+^ and CD8^+^CD70^+^ T cells (H) in blood (PB) and skin. (I) Serum levels of sCD27 measured at day 14 after SCT in patients who subsequently developed aGVHD (n = 22), compared with patients who never developed aGVHD (n = 11). (J) Serum sCD27 measured in patients at onset of GVHD (n = 25), compared with patients who never developed aGVHD (n = 15) at matched time points after HSCT. Statistical comparisons were made using Wilcoxon matched-pairs signed-rank test (A-E,G): ∗*P* < .05; ∗∗*P* < .01; ∗∗∗*P* < .005. FITC, Fluorescein isothiocyanate.

Considering the early activation of CD70^+^ T cells after transplant, likely in response to allogeneic stimulation, our focus shifted to understanding their potential correlation with subsequent development of GVHD. Thirty-five percent of patients developed aGVHD (grade 2-4) at a median of 50 days after allo-SCT and regular prospective sampling allowed for us to acquire blood samples within 4 days before the onset of aGVHD. The relative expression of CD70^+^ T cells at this time was compared with the values within samples from matched time points in patients who did not develop aGVHD. The percentage of CD4^+^CD70^+^ and CD8^+^CD70^+^ T cells was 9.6-fold and 4.2-fold higher in patients with severe aGVHD (25% and 13%; Figure 4A) than patients with no GVHD (2.6% [*P* < .005] and 3.1% [*P* < .05], respectively). In patients with aGVHD, CD4^+^CD70^+^ T cells further demonstrated heightened intracellular expression of inflammatory and cytotoxic proteins, including IFN-γ, interleukin-17, perforin, and granzyme (Figure 4B-E). We also observed more circulating CD4^+^CD70^+^ T cells in patients with chronic GVHD (median onset 210 days after transplant; supplemental Figure 4A).

We further assessed the trafficking of CD70^+^ T cells to GVHD grade 2 to 4–affected tissues using immunofluorescence imaging of diagnostic biopsies of skin, gut (rectum or sigmoid), and liver tissues (n = 6; Figure 4F). CD3 and CD70 coexpressing cells could be detected in all tissues in close proximity with tissue damage. We further quantified CD70^+^ T cells in collagenase digested skin biopsies taken at GVHD diagnosis. Notably, CD8^+^CD70^+^ T cells here exhibited an 11-fold increase (18%) compared with healthy skin (2%; *P* < .05), whereas CD4^+^CD70^+^ T cells were 2.6 times more prevalent (11% vs 4%; supplemental Figure 4B). CD70^+^ T cells within tissue exhibited higher expression of chemokine receptors than CD70^+^ T cells circulating in blood. In particular, 81% of CD4^+^CD70^+^ T cells within tissue expressed CCR4, 63% cells expressed CCR6, and 50% cells expressed both receptors, whereas in blood, these values were 17%, 7%, and 3%, respectively (Figure 4H). Analysis of CD8^+^CD70^+^ T cells in tissue showed that 53% were CCR4^+^, 22% were CCR6^+^, and 19% were CCR4^+^CCR6^+^ compared with 10%, 17%, and 6%, respectively, in blood (Figure 4G).

CD27 is the ligand for CD70, and receptor engagement can lead to the release of soluble CD27 (sCD27). The percentage of CD27^+^ T cells in blood was not associated with GVHD (supplemental Figure 4C). However, sCD27 level at day 14 was predictive of subsequent risk of GVHD (median 43 IU/mL in patients who subsequently developed aGVHD vs 36 IU/mL in those who remained free of GVHD; *P* < .05; Figure 4I). Furthermore, sCD27 levels were further increased at the onset of GVHD (grade 2-4) at 107 IU/mL compared with 74 IU/mL in patients with low-grade or no aGVHD at a matched time point (Figure 4J).

CD70^+^ T cells demonstrate alloreactive specificity in vitro, and functional responses are reduced markedly by CD70 blockade

**Figure 5.**
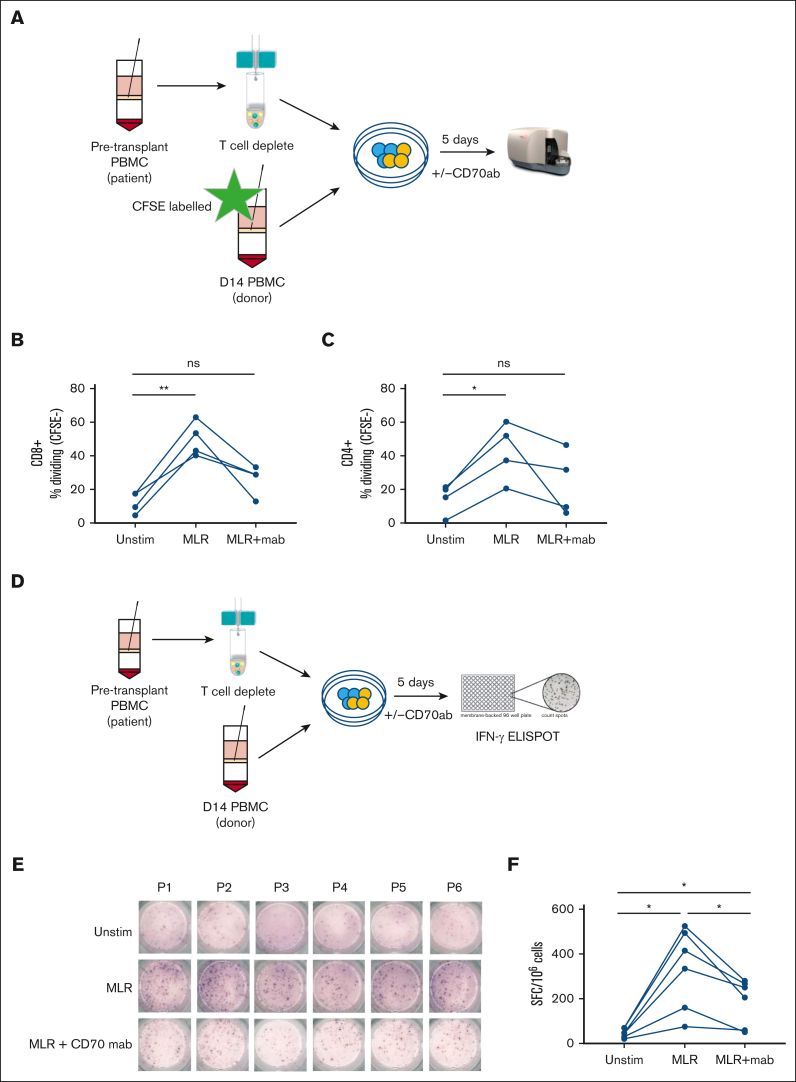
**Blocking of CD70^+^ decreases proliferation and IFN-γ secretion in donor T cells.** (A) Schematic view of the proliferation assay performed on day 14 patient PBMCs (n = 6; 5 MUD and 1 sibling donor) after allo-HSCT and stimulated with recipient patient’s T-cell–depleted pretransplant PBMCs in the presence/absence of anti-human CD70 antibody. (B-C) Percentage of CFSE-CD8^+^ T cells (B) and CFSE-CD4^+^ T cells (C) measured after 5 days with no stimulation (unstim), in a mixed lymphocyte reaction (MLR) with isotype control, and in MLR with anti-CD70 antibody. (E) Schematic view of the IFN-γ secretion assay performed on day 14 PBMCs from allogeneic transplant patients (n = 6; 5 MUD and 1 sibling donor) in the presence/absence of anti-CD70 antibody. (E) IFN-γ secretion measured by ELISPOT for the various direct stimulation methods measured PBMCs of 6 patients who underwent allo-HSCT in the presence/absence of CD70 antibody. (F) Frequency (mean) of IFN-γ SFC per 1 × 10^6^ PBMCs after 5 days of stimulation followed by an overnight ELISPOT as indicated (n = 6; ∗*P* < .05). Statistical comparisons were made using 1-way ANOVA with Tukey multiple comparison test: ns, not significant; ∗*P* < .05; ∗∗*P* < .01. CFSE, Carboxyfluorescein succinimidyl ester; mab, monoclonal antibody; MUD, Matched Unrelated Donor.

To evaluate the functional impact of CD70 blockade on alloreactive recognition in vitro, T cells isolated from blood at day 14 after transplant were cocultured with pretransplant T-cell–depleted patient PBMCs in the presence or absence of anti-CD70 monoclonal antibodies (Figure 5A). Alloantigen stimulation increased the CD4^+^ and CD8^+^ T-cell proliferation by 28% and 38%, respectively, compared with donor T cells alone. Of note, these values were reduced by 19% and 24%, respectively, in the presence of anti-CD70 antibodies (Figure 5B-C). Cytokine secretion was further evaluated using an IFN-γ ELISPOT assay after 5 days of culture with or without allogeneic stimulation (Figure 5D). Spontaneous IFN-γ secretion, without stimulation, was seen in 56 cells (spot forming units [SFCs]) per 1 × 10^6^ PBMCs, increasing nearly eightfold to 443 SFCs per 1 × 10^6^ PBMCs after stimulation with patient’s antigen presenting cells. Importantly, this was reduced by 44% in the presence of anti-CD70 treatment to 250 SFCs (Figure 5E-F).

## Discussion

T-cell–mediated alloreactive responses underpin clinical outcomes after allo-HSCT, and here, we identify CD70 as an early marker of alloreactive T cells. The findings reveal potentially important insights into the pathophysiology and management of aGVHD.

The number of CD70^+^ T cells increases rapidly after transplantation and peaks at around day 14 after allo-HSCT. CD70 expression is potently induced by alloantigen,^18^ but this expression is not observed after incubation with homeostatic cytokines.^11^ Nevertheless, it remains unclear whether intense homeostatic proliferation after transplantation could contribute to CD70 expression. However, our finding of expression of CD95 (Fas cell surface death receptor) on CD70^+^ T cells is indicative of recent TCR engagement and, together with functional alloreactive specificity in vitro, suggests that this is likely to be driven by the recognition of minor histocompatibility peptides. This temporal pattern aligns with the onset of alloreactive immune recognition, demonstrated most clearly in the efficacy of cyclophosphamide-mediated abrogation of aGVHD at day 4 after transplant.^27^ CD70^+^ T-cell numbers remained higher on CD4^+^ subsets,^7^ and this is noteworthy given the importance of CD4^+^-mediated alloreactive responses as demonstrated by the tumor cell HLA class II downregulation leading to leukemia relapse.^28^

The biological importance of CD70 expression on activated T cells is uncertain. CD70 is expressed an antigen presenting cells, and it is possible that T cells acquire this phenotype to activate additional T cells at sites of inflammation. This “T-cell costimulation” may be reflected in the expression of CD80 and CD86, together with costimulatory molecules CD28, ICOS, and CD154, on CD70^+^ T cells.^29^ Expression of checkpoint proteins PD-1 and LAG3 was also seen on CD70^+^ T cells, and although strong type 1 helper T cell (Th1) cytokine and cytotoxic phenotype suggests little functional exhaustion, this may explain the increased risk of GVHD associated with posttransplant PD-1 inhibition.^30^

ATAC-seq analysis provided insights into the chromatin landscape and transcriptional regulation during transition to CD70^+^ T cells. No differential chromatin accessibility was observed at the CD70 or CD27 loci, emphasizing the potential role of promoter methylation.^31^ TF binding site analysis showed closure at the MGA locus, an antiproliferative TF that competes with Myc for binding to Max,^32^ during transition to CD70^+^ state, whereas the T-cell transcriptional regulators *POU2F1* (Oct1) and POU5F1:SOX2^33^ became more accessible.^34^ *POU2F1* interacts closely with c-Myc to regulate cell cycle progression,^35^^,^^36^ and prior reports have shown that inhibition of Myc pathways can prevent GVHD while preserving graft-versus-leukemia.^37^ Interestingly, *NFAT5* transactional activity, pivotal for Th17 polarization, was also enriched in CD70^+^ T cells.^38^ Transcriptional studies confirmed the activated and proliferative nature of CD70^+^ T cells. Enrichment of MAPK signaling suggests that CD70^+^ T cells use both TCR and CD70 to activate the NF-κB pathway, whereas the Toll-like receptor expression indicates differential pathogen sensing.^39^^,^^40^ CD70^+^ T-cell populations showed dominant clonotypes, and the oligoclonal profile was similar to CD70^–^ cells. Clonal overlap of TCR use was seen between CD70^+^ and CD70^–^ subsets within individual patients and indicates that alloreactive T cells are also likely to reside within the CD70^–^ pool.

A striking increase in the percentage of CD4^+^ and CD8^+^CD70^+^ T cells was seen immediately before the onset of aGVHD, particularly for Th1 CD4^+^CD70^+^ cells, and suggests an active role in GVHD pathogenesis. Importantly, elevated concentrations of soluble CD27, released after CD27-CD70 engagement,^41^^,^^42^ were seen at day 14 in patients who subsequently developed aGVHD and highlight the potential utility of sCD27 as a biomarker for aGVHD prediction. CD70^+^ T cells express tissue homing receptors CCR4 and CCR6^43^ and were enriched in aGVHD tissue. Future spatial analyses will be of interest to assess relative contribution to inflammation in different tissue subtypes.

CD70^+^ T cells demonstrated alloreactive specificity in vitro, and CD70 blockade decreased effector cell proliferation and cytokine release by 24% and 19%, respectively, along with a reduction in IFN-γ release. This augurs well for the potential of therapeutic CD70 blockade to suppress aGVHD, although the relative clinical impact of this degree of suppression will need assessment. It should also be noted that TCR sequences were shared between CD70^+^ and CD70^–^ subsets, and as such, some alloreactive clones may remain within the CD70^–^ pool. CD70 blockade might also be considered as a prophylactic measure to prevent the generation of aGVHD, although here, it will be important to determine that the graft-versus-leukemia effect is not substantially attenuated. Of note, CD70 is itself expressed by leukemic blasts in acute myeloid leukemia^44^ and as such may represent an ideal target in this disease subtype.^45^

In future studies, it will be important to study the impact of differential conditioning regimens on the induction of CD70^+^ T cells and the relative homing of these subsets to different tissue sites. Furthermore, although studies in murine models have shown that CD70 blockade can reduce solid organ graft rejection,^18^ CD70 deletion in host cells enhanced GVHD in a major histocompatibility complex–mismatched murine model.^17^ Limitations of our study include low T-cell number recovery at early time points after SCT that restricted the diversity of functional assays.

Our study defines the kinetics, functional correlates, and clinical associations of CD70 expression after transplantation. These observations not only provide insights into aGVHD pathogenesis but also support therapeutic targeting of CD70 as a promising prophylactic and therapeutic strategy to mitigate this challenging clinical condition.

Conflict-of-interest disclosure: The authors declare no competing financial interests.
