# Supplementary material for: CD70 identifies alloreactive T cells and represents a potential target for prevention and treatment of acute GVHD
Source: Blood Adv. 2024 Jul 23;8(18):4900–12. doi: 10.1182/bloodadvances.2024012909 (PMC11421336; doi:10.1182/bloodadvances.2024012909)
Supplement: Supplemental Figures, Tables, and Methods [file BLOODA_ADV-2024-012909-mmc1.pdf]

Supplemental Data

Supplemental Figures

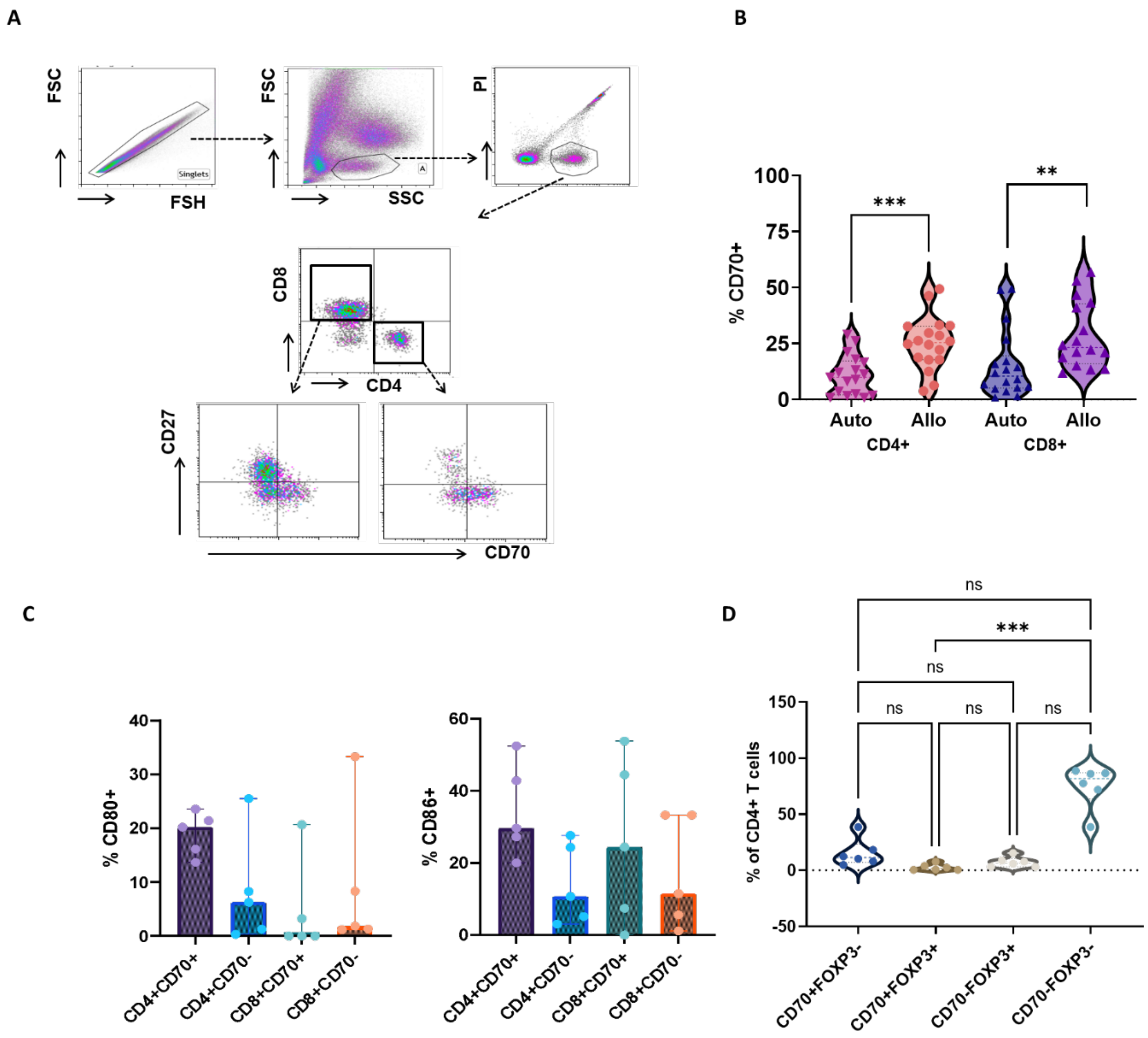

Supplemental Figure 1. (A) Representative flow cytometry gating plot from patient sampled at +14 days post-SCT (B) Percentage of CD8+CD70+ and CD4+CD70+ T cells in patients who received autologous (n=18) or allogeneic SCT(n=16) measured at two weeks post-transplant. (C) Percentage CD80+ and CD86+ expression on CD4+CD70+/- and CD8+CD70+/- T cell populations. (D) Percentage of FOXP3+ cells within the CD4+CD70+ and CD4+CD70- T cell population. Statistical comparisons were made using Mann-Whitney test (B) and one-way ANOVA with Dunn's Multiple Comparison test (C); Wilcoxon matched-pairs signed rank test (D) * - p<0.05. ** - p<0.01. *** - p<0.005.

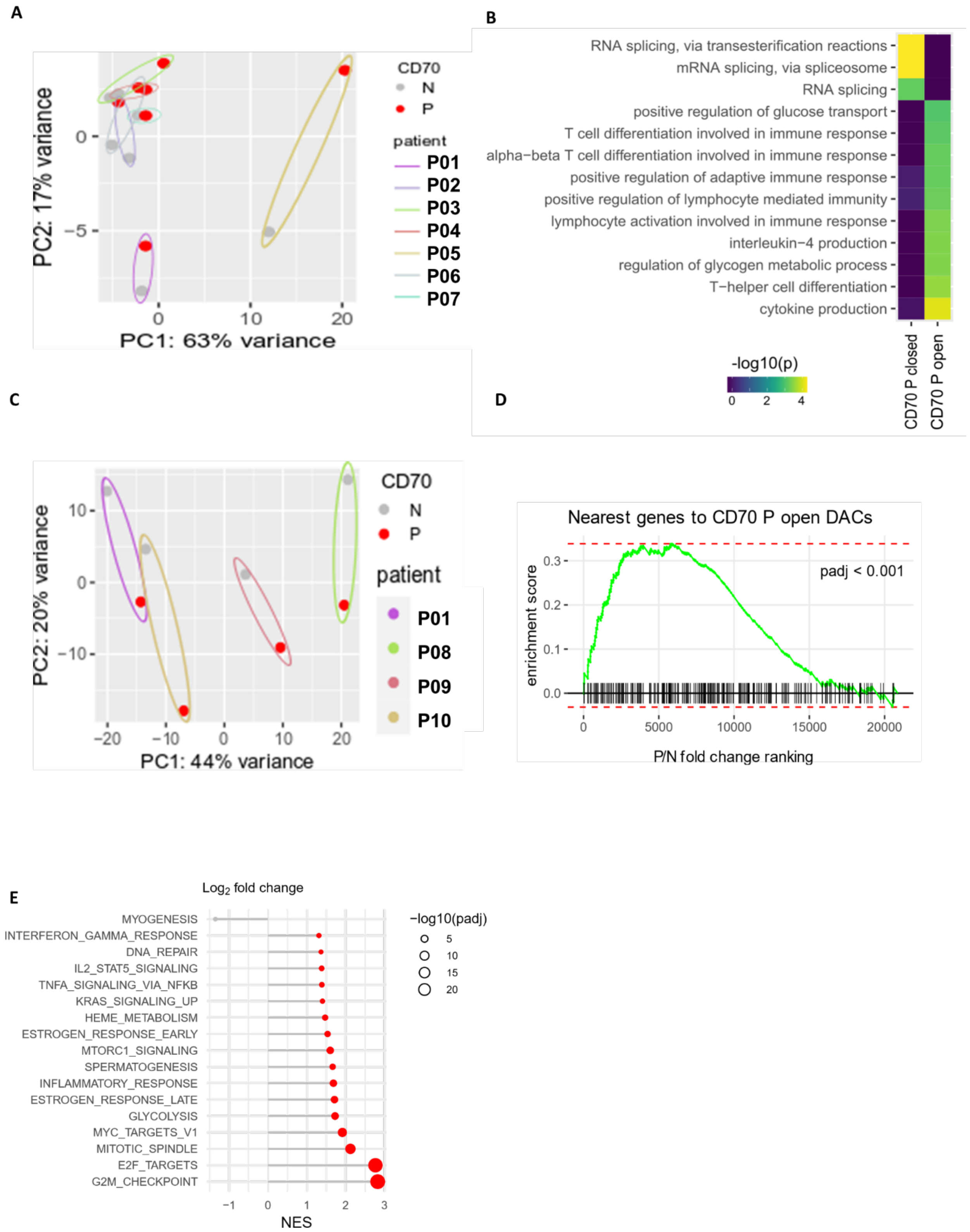

Supplemental Figure 2. (A) PCA plot depicting percentage variance of the peak counts between CD70+(red) and CD70- (grey) chromatin for each sorted patient sample. (B) GO-term biological process enrichment analysis determines which biological processes are enriched within CD70+ vs CD70- DAC regions. (C) PCA plot depicting percentage variance of the gene counts between CD70+(red) and CD70- (grey) within each sorted patient sample. (D) GSEA integrates the

chromatin accessibility data and gene expression data and explores the correlation between chromatin structure and gene regulation (p<0.001). (E) Gene set enrichment analysis (GSEA) using genes ranked by log2FC in CD70+ vs CD70- cells. Visualized are Hallmark gene sets with adjusted p-value < 0.1 and positive NES indicates enrichment towards expression in CD70+ samples.

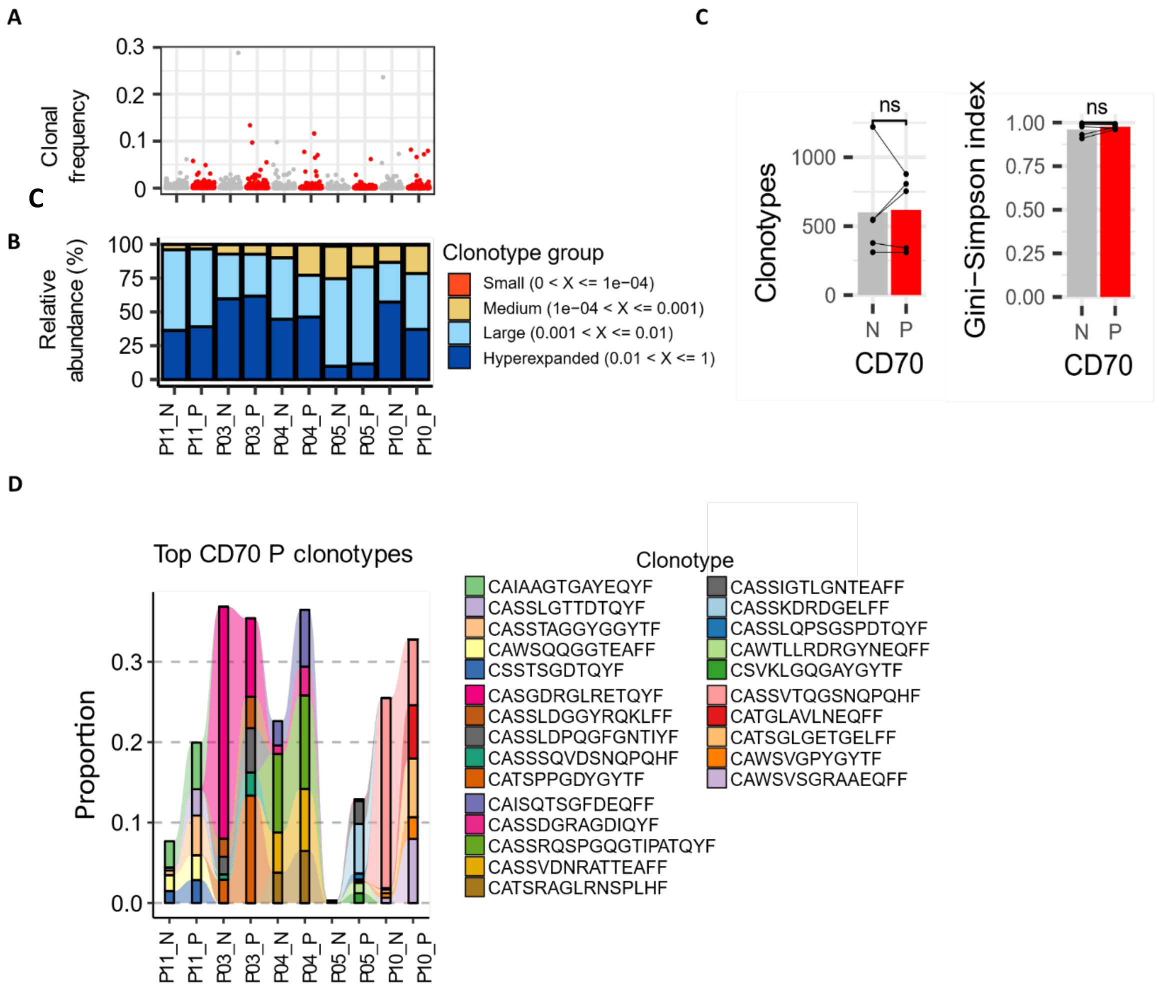

Supplemental Figure 3. (A) Overall clonotype frequency and (B) Relative abundance of small to hyperexpanded clonotypes within CD70 + (P) and CD70- (N) cells for each patient sample. (C) Bar plots comparing of number of identified clonotypes (left) and clonotype diversity using Gini-Simpson index (right) between N and P cell populations. (D) Relative proportion and sequence of dominant hyperexpanded CD70+ Clonotypes.

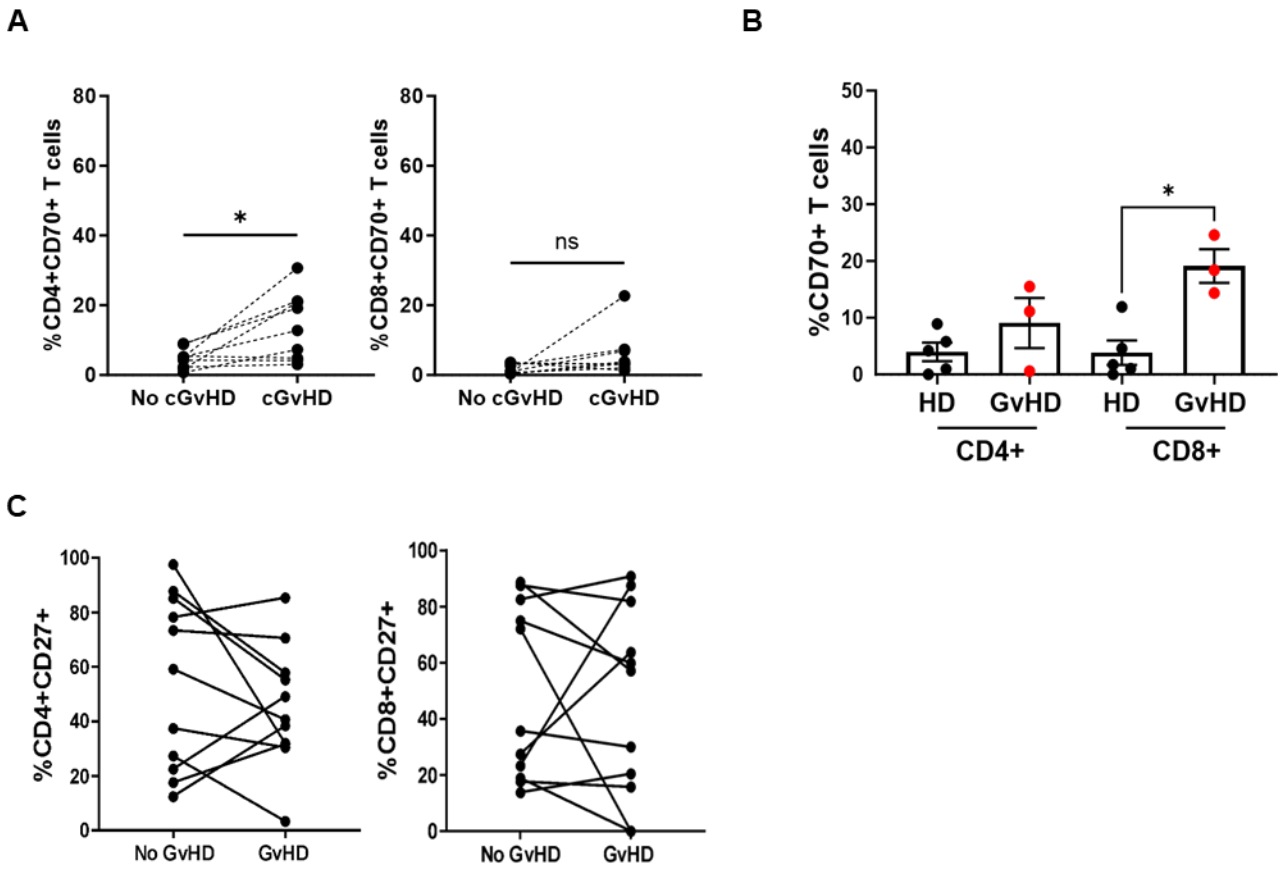

Supplemental Figure 4. (A) Percentage CD70+ T cells within the CD4+ or CD8+ T cell compartment in patients with chronic GvHD measured within 0 -7 days of diagnosis compared to patients who did not develop GvHD, measured at matched time points post-transplant (median = 210 days post-transplant, n=9 each cohort) (B) Percentage of CD70+ cells within the CD8+ and CD4+ T cell compartment in skin of patients with aGvHD (n=3) measured by flow cytometry in digested skin biopsies collected at aGvHD diagnosis compared to skin of healthy donors (HD, n=5). (C) Percentage of CD4+CD27+ T and CD8+CD27+ cells in allogeneic SCT patients at aGvHD diagnosis vs those who did not develop aGVHD (No GvHD) at matched time-points. Statistical comparisons were made using Wilcoxon matched-pairs signed rank test; * - $p<0.05$. ** - $p<0.01$. *** - $p<0.005$.

Supplemental Methods

Supplemental Table 1. List of antibodies used for phenotypic characterisation of CD70+/- T cells

Target ab	Clone	Company	Catalogue No.
CD3	OKT-3/HIT-3a	Biolegend	317340/300326
CD4	RPA-T4	Biolegend	300517
CD70	113-16	Biolegend	355110
CD25	M-A251	Biolegend	356108
CD28	CD28.2	Biolegend	302920
ICOS	C398.4A	Biolegend	313506
CTLA-4	BNI3	Biolegend	369614
CD154	24-31	Biolegend	310820
CCR6	G034E3	Biolegend	353424
CCR4	I.291H4	Biolegend	359414
CD80	W17149D	Biolegend	375406
CD86	IT2.2	Biolegend	305422
PD-1	EH12.2H7	Biolegend	329914
CCR7	4B12	Biolegend	120108
DNAM-1	11A8	Biolegend	338304
IL-17A	BL168	Biolegend	512318
PERFORIN	B-D48	Biolegend	353304
IFNγ	4S.B3	Biolegend	502512
KI67	KI-67	Biolegend	350526
LAG-3	11C3C65	Biolegend	369310
CD45RA	HI100	Biolegend	304120
CD8	BW135/80	Miltneyi	130-113-164
CD95	DX2	eBiosciences	12-0959-42
CD27	M-T271	BD Pharmingen	555441
GRANZYME B	GB11	BD Pharmingen	560211
CD14	RMO52	Beckman Coulter	IM2707U
CD19	J3-119	Beckman Coulter	A07770

Supplemental Table 2. Patient characteristics of autologous patients included in analysis of Supplementary figure 1B

Characteristic	Feature	Autologous tranplant
Total		N=18
Age	Median (Range)	56 (16-72)
Gender	Male	14
	Female	4
Disease	Myeloma	9
	Hodgkins Lymphoma	4
	Non Hodgkins Lymphoma	1
	Mantle cell Lymphoma	2
	Anaplastic Large Cell Lymphoma	2
CMV	Negative	6
	Positive	12
Conditioning	Melphalan	9
	BEAM	8
	LEAM	1
	TBI	0
Relapse	n (median day onset post transplant)	5 (269)

ATAC seq Data Analysis

Raw reads were quality checked using FastQC v0.11.9 and multiQC v1.9. Reads were trimmed to remove nextera adapter sequences using Trim_Galore v0.6.5 with default parameters. Trimmed reads were aligned to the human genome reference GRCh38.genome.fa using Bowtie2 v2.3.5 (parameters: –very-sensitive-local). Alignments were filtered to keep only high quality (>q30) aligned reads from Chr1-22, ChrX and ChrY using Samtools v1.10 and duplicates removed using picard v2.21.1. Peaks were called on the filtered alignments for each sample using MACS2 v2.2.7 with settings: --keep-dup=auto –nomodel –shift -100 –extsize 200. Peak summit files were filtered to remove peaks in known blacklisted regions (ENCFF356LFX_grch38_blacklist.bed). A union peak set across all samples was constructed using BEDtools v2.30. The union peak set was used as the master reference peak loci to calculate the peak by sample count matrix using the annotatePeaks function from HOMER v4.11. This function also annotated peaks with closest gene (by RefSeq transcription start site) and associated genomic feature. Count data was normalised and CD70+ vs CD70- differentially accessible chromatin (DAC) loci were identified using the R package DESeq2. To identify CD70+ vs CD70- specific effects, paired design which includes a patient identifier in the design formula (patientID ~ cd70) was used in order to account for additional covariates amongst patients.

RNA seq Data Analysis

Raw reads were processed with the Dragen pipeline for quality checking and mapping to the human genome version hg38_alt_aware. Aligned reads were quantified at the gene expression level using HTSeq to produce per-sample feature count matrices. Raw counts were filtered to remove genes that had no expression across all samples. DESeq2 was used for normalisation and differential expression comparison of CD70+ vs CD70- sample groups. Genes were considered to be differentially expressed if Benjamini Hochberg adjusted p-value<0.05 and absolute log2 fold change >0.5. To identify CD70+ vs CD70- specific effects, paired design which includes a patient identifier in the design formula (patientID ~ cd70) was used in order to account for additional covariates amongst patients.

Immune Repertoire data analysis

Raw reads were quality checked using FastQC v0.11.9 and multiQC v1.9. MiXCR v4.3.2(20) was used to align to human TRA and TRB reference sequences, and assemble TCR clonotypes using *mixcr analyze qiaseq-human-tcr-cdr3*. TCRB CDR3 clonotypes from MiXCR were further analysed and

visualised in R with the Immunarch package. To avoid the potential influence of sequencing depth on the analysis of clonotype frequency and diversity, the TCR repertoires of all the individuals involved were downsampled to the same size as the smallest repertoire (272,543 TCR sequences per sample). Clonotype counts, abundance distributions, diversity and repertoire overlap were assessed using Immunarch functions repExplore(), repDiversity() and repOverlap. To assess frequencies of clonotypes against antigens, clonotypes were matched against the public TCRB CDR3 amino acid sequences in the VDJ TCR database and matches were annotated with known antigen specificity. To identify CD70+ vs CD70- specific effects, paired design which includes a patient identifier in the design formula (patientID ~ cd70) was used in order to account for additional covariates amongst patients.
